# A Rare Case of Recurrent Bowel Obstruction: Midgut Malrotation in an Adult Patient

**DOI:** 10.1155/cris/8979342

**Published:** 2026-08-02

**Authors:** Andreas Koumenis, Nikoleta Sinou, Kyriaki Karafotaki, Evangelia Dimitrakopoulou, Evgenia Mainta, Dimitrios Filippou, Georgios Noussios, Ioannis Kaklamanos, Theodoros Mariolis-Sapsakos

**Affiliations:** ^1^ University Surgical Clinic, General and Oncological Hospital of Kifisia “Oi Agioi Anargiroi”, Athens, Greece; ^2^ Department of Anatomy, School of Medicine, NKUA, Athens, Greece, uoa.gr; ^3^ Member of Hellenic Academic Libraries Link-HEAL (Link), Thessaloniki, Greece; ^4^ Radiology Department, General and Oncological Hospital of Kifisia “Oi Agioi Anargiroi”, Athens, Greece; ^5^ Department of Anatomy, School of Physical Education and Sport Sciences, AUTh, Serres, Greece; ^6^ Anatomy, Embryology and Histology Lab, Faculty of Nursing, NKUA, Athens, Greece, uoa.gr

**Keywords:** bowel obstruction, congenital abnormality, midgut malrotation

## Abstract

Midgut malrotation (MM) is a rare congenital abnormality that is usually diagnosed early in life during the neonatal period or the first year of life. In some rare cases, the patient may remain asymptomatic or with only mild nonspecific symptoms that delay the diagnosis until late adulthood. These nonspecific gastrointestinal symptoms can culminate in an adhesive bowel obstruction or volvulus making the presentation a surgical emergency. In our case, we present a 73‐year‐old male with nonspecific symptoms that led to the diagnosis of adhesive obstruction of the duodenum from Ladd bands due to undiagnosed MM. A laparotomy with Ladd’s procedure was performed.

## 1. Introduction

Midgut malrotation (MM) is a range of congenital disorders that are caused by the midgut’s partial or total inability to rotate 270° counterclockwise around the superior mesenteric vessels during fetal development. The incidence is around 1 in 6000 newborns, with most of these cases becoming symptomatic during the first month of life and a further 90% up to 1 year after birth; however, some cases remain asymptomatic well into adulthood. Adult presentation is reported to be as low as 0.2% of the population [1, 2].

In this case report, we present an adult male patient with a history of intermittent discomfort after food and recurrent episodes of bowel obstruction who was diagnosed late in adulthood with MM. This case report highlights the atypical clinical presentation of this report, the clinical investigations that were made, and the surgical management.

## 2. Case Presentation

A 73‐year‐old male presented to our Emergency Department with complaints of periumbilical and epigastric colicky pain, the onset of which was 10 h ago and is gradually worsening. He also mentions two episodes of bilious vomiting in the last 4 h. From his personal history, he states recurring episodes of nausea with or without vomiting as well as feelings of early satiety and periodic abdominal discomfort after meals. These symptoms of colicky pain and vomiting were present irregularly for the past 5 years but were discarded by the patient as nonurgent, and did not seek medical advice. He mentions no changes in bowel habits and no fever. He has no past surgical history, and his past medical history consists of hypertension, hypothyroidism, and dyslipidemia.

The patient had normal vitals with 123/72 mm Hg blood pressure, 119 bpm pulses, and a body temperature of 36.8°C. A review of the other systems was normal. Blood tests revealed a normal leucocyte count, a hemoglobin of 13.2 g/dL, and 420 × 10^9^/L platelets. BUN, serum electrolytes, and creatinine were all within range.

Physical examination revealed abdominal pain on deep palpation of the left upper quadrant as well as the periumbilical area and abdominal distention. Bowel sounds were present, and a digital rectal exam was normal. An erect abdominal X‐ray was ordered that revealed air‐fluid levels mainly in the left upper quadrant. Due to the air‐fluid levels, a CT scan was also ordered.

**Figure 1:**
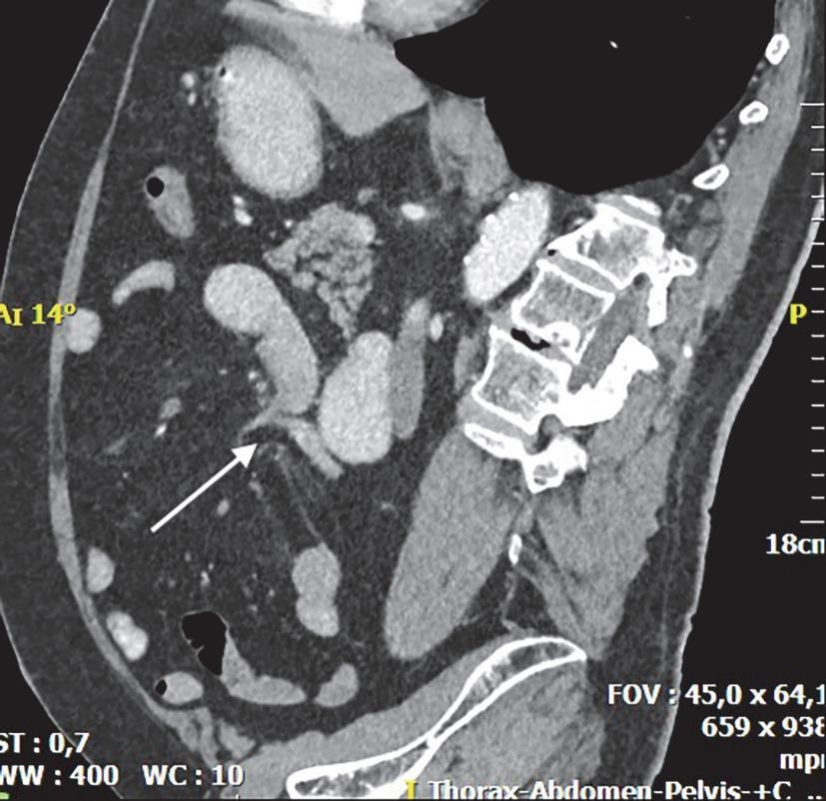
The “wirlpool” sign.

CT revealed an ectopic position of the cecum and appendix in the lower left quadrant and a “folding” of the 2nd and 3rd parts of the duodenum near the Treitz ligament. Cephalic to the ligament, the duodenum and stomach were dilated. A “whirlpool sign”—a twist of the mesentery around the superior mesenteric artery (SMA)—is also visible as well as multiple duodenal adhesive bands that cause the extrinsic duodenal obstruction (Figure 1). These findings were highly suspicious of adhesive bowel obstruction due to MM.

The patient was admitted to the clinic and managed with a nasogastric tube decompression—which drained around 200cc of bilious gastric fluid—and intravenous fluid hydration. After informing the patient of the diagnosis, he consented to a definitive treatment of the duodenal obstruction, so surgery was scheduled. The patient underwent exploratory laparotomy that revealed the cecum and colon contained in the left abdomen and the entire small bowel in the right. Multiple Ladd’s adhesions were also present at the 2nd and 3rd parts of the duodenum. The surgeons began the Ladd’s procedure, performing mobilization of the duodenum with adhesiolysis and straightening of the C‐loop. The adhesiolysis was continued, and any adhesions surrounding the SMA were also lysed. Last, an appendectomy was performed.

The patient’s recovery was uneventful, with first flatus on the 2nd postoperative day, and was discharged 2 days later, on postoperative day 4. As of now, 1 year postoperatively the patient is without any recurrence of abdominal pain.

## 3. Discussion

Intestinal malrotation is a spectrum of congenital conditions deriving from the failure of the intestine to rotate properly to its final position during embryogenesis. Around the 5th week of gestation, the midgut, which until then follows a vertical path parallel to the SMA axis, is outgrowing the abdominal cavity and is forced to herniate into the developing umbilical defect along with its blood supply—the SMA. Inside this herniated position, the midgut’s first rotation takes place. Between weeks 5 and 10, a 90° counter‐clockwise rotation around the axis of the SMA takes place, placing the duodenojejunal loop (cephalic end of midgut) to the right of the SMA and the ceco‐colic loop to the left. The intestinal tube is then retracted back into the abdominal cavity, and the second stage happens between the 11th and 12th weeks of embryogenesis where a further 180° rotation (for a total rotation of 270°) takes place so that the duodeno‐jejunal loop is placed to the left of the SMA and the ceco‐colic loop to the right and superior to the SMA. Consequently, the small bowel and duodenal “C” loop adhere to the posterior wall, with the descending colon to the left, the transverse colon above, and the ascending colon to the right. The third and final stage occurs from the 12th week onwards and involves the fixation of the mesentery, placing the ceco‐colic loop to the right of the SMA.

Depending on the extent to which the bowel fails to follow these normal rotational stages, different classifications of MM arise. It can cause a shorter mesenteric root and a more narrow suspensory pedicle (ligament of Treitz) in the bowel. The small bowel can warp around the narrowed ligament due to the short mesentery, thus putting the patient at risk of midgut volvulus and subsequent intestinal ischemia.

**Figure 2:**
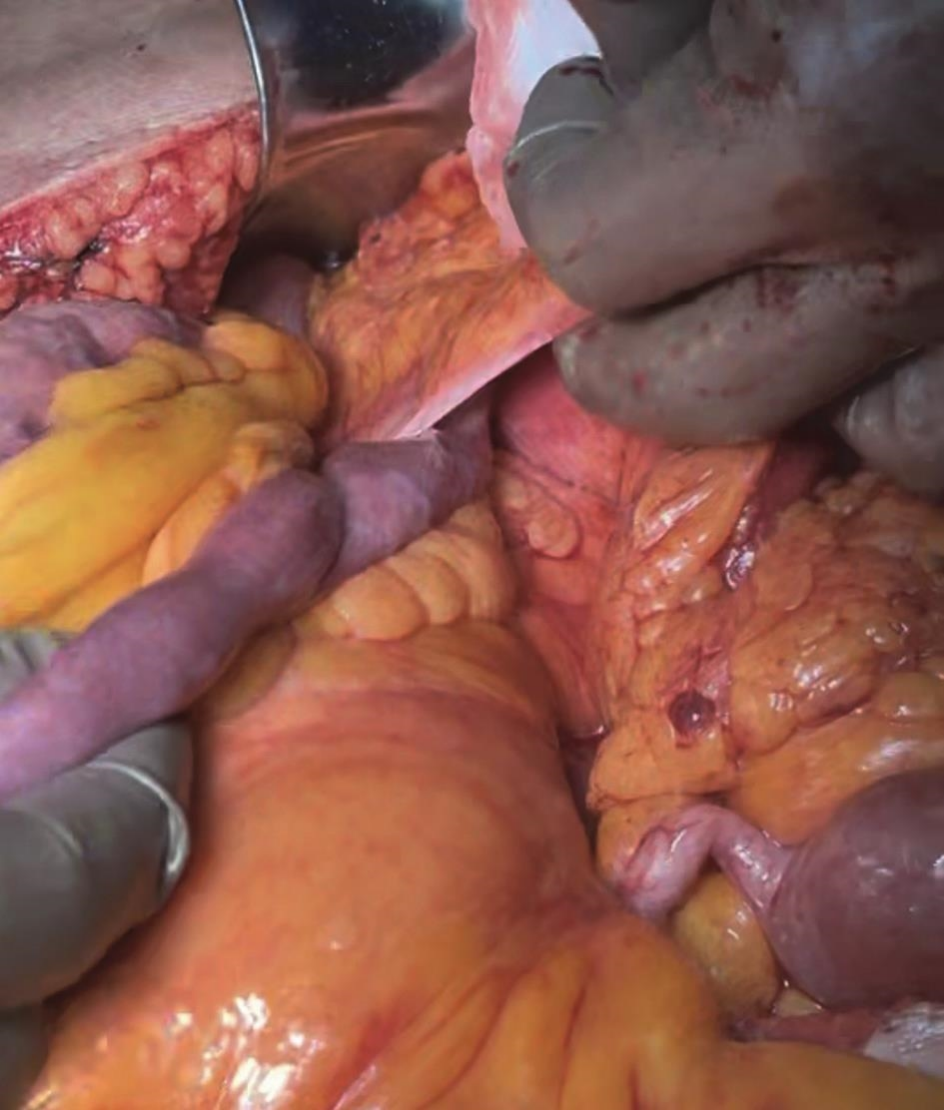
Fibrous Ladd’s adhesive bands of the duodenal pre‐adhesiolysis.

Even if no acute onset of volvulus presents, congenital adhesive bands known as Ladd’s bands can cause chronic obstruction of the duodenum—as in the case of our patient (Figure 2) [3].

Generally, this abnormality can result in two major types of malrotation: an incomplete rotation or a nonrotation. In incomplete rotation, there is no more than a 180° rotation of the cranial or the caudal portions. As a result, the cecum is situated immediately in front of the SMA, and the proximal midgut is positioned to the right of the SMA. This has Ladd’s bands covering and encroaching on the anterior duodenum. In the nonrotation type, the proximal midgut is fixed anterior to the right of the SMA and the cecum anterior to the left because neither component rotates more than 90°. The mesentery is, as expected, shorter and narrower [4].

Although the exact etiology of the malrotation is unknown, some studies suggest that a mutation in FOXF1 (forkhead box transcription factor) and L‐R asymmetry genes may be present [2].

As already mentioned, most of the cases present very early in life, in neonates, but asymptomatic variations may present with very late‐onset symptoms in adulthood.

The main symptoms are usually nonspecific, making the diagnosis even more challenging. In adults, it may present as an acute abdomen requiring urgent intervention, or it may present in the nonacute setting with chronic, nonspecific symptoms like abdominal pain, distention, vomiting, and food intolerance. These ill‐defined abdominal complaints are usually present since childhood and are often misdiagnosed as peptic ulcer disease, irritable bowel syndrome, pancreatic and biliary diseases, and psychiatric disorders. In the literature, adult presentation of MM can be divided into two subgroups: (a) the acute onset group, which will have obstructive symptoms with signs of acute abdomen and disclose a prior history of sparse, but milder symptomatology upon questioning, and (b) as in our case, the patient may present with chronic symptoms of bowel obstruction and a history of recurring episodes of nausea, early satiety, crampy abdominal pain, and bilious vomiting. They may also mention weight loss, hematochezia, headache, and change in bowel habits [5].

An abdominal X‐ray during the initial investigation may reveal a gasless abdomen with large bowel gas on the right or occasionally a “double‐bubble” sign—a sign of dilatation of the proximal duodenum and stomach due to duodenal obstruction [6].

Upper gastrointestinal imaging series (UGI) is thought of as the “gold standard” in the pediatric population, with oral contrast being administered to capture the passing to the duodenal‐jejunal junction (DJJ) with images. Atypical anatomical positions of the DJJ to the left of or overlying the spine and below the level of the duodenal bulb, low DJJ position, the right jejunum, and a DJJ corkscrew appearance (in the event of volvulus) are suggestive of MM. However, even though the duodenojejunal loop may rotate normally during embryogenesis, the cecocolic loop can still independently fail to normally rotate, and as a result, a normal UGI does not rule out malrotation.

Nevertheless, a normal UGI does not exclude the possibility of malrotation, as rotation abnormalities of the cecocolic loop may occur even with a normal rotation of the duodenojejunal loop.

In adults, an abdominal CT scan is preferred and can set the diagnosis if a pathognomonic radiological sign referred to as the “whirlpool” sign, caused by the small bowel swirling around the SMA, is present. Also, the SMA and the superior mesenteric vein (SMV) can be seen inverting sides as well as a right‐sided small bowel, a left‐sided cecum, and aplasia of the uncinate process of the pancreas [7].

If an upper GI fluoroscopy is performed, other signs may be visible, like the “swirl sign,” “bird’s beak” or “corkscrew” sign. A study by Fu et al. [5], which included nine patients diagnosed with MM, stated that in only three of the five patients that underwent a CT scan, the diagnosis was set, emphasizing that the untrained eye may overlook the atypical finding.

**Figure 3:**
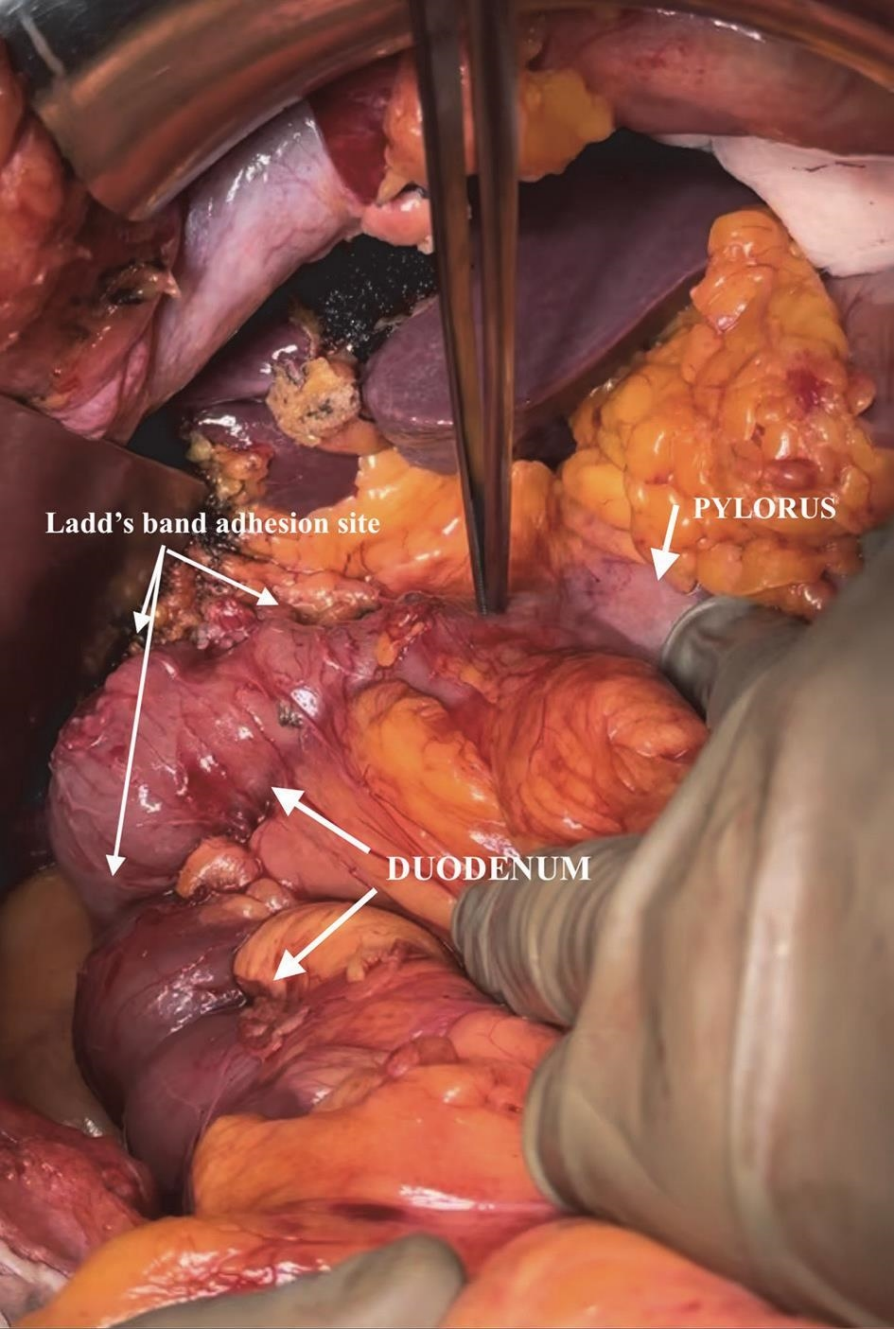
The duodenum fully mobilized after dissection and adhesiolysis of Ladd’s bands.

Surgical intervention is the only defined treatment for the intermediate or complete obstruction caused by MM. In the classic Ladd’s procedure, the volvulus, if present, is reversed, the duodenum is mobilized by dividing the abnormal colo‐duodenal Ladd’s bands, which tether the midgut and cause external compression, and the adhesions around the SMA are divided to allow for a broader mesenteric base in an attempt to prevent further volvulus (Figure 3). An appendectomy also takes place since the abnormal position of the cecum and appendix post‐Ladd’s procedure makes the diagnosis of acute appendicitis more difficult.

## 4. Conclusion

MM is a rare cause of acute or chronic abdominal pain in adults, deriving from the abnormal rotation of the midgut during embryonic development. Even though symptoms are usually evident very early in life, nonsymptomatic or mildly symptomatic cases can go unnoticed until well into adulthood with nonspecific symptoms, making the diagnosis difficult. UGI is the gold standard for diagnosis, although a CT scan can also be diagnostic and is more readily available. Ladd’s procedure, either laparoscopically or with laparotomy, is the traditional treatment. Our case highlights this rare entity in adult patients with nonspecific symptoms, stressing the importance of having a high index of suspicion for malrotation regardless of the patient’s age so a definitive treatment can be given.

## Funding

The publication of the article in OA mode was financially supported by HEAL‐Link.

## Conflicts of Interest

The authors declare no conflicts of interest.

## Data Availability

The data that support the findings of this study are available upon request from the corresponding author. The data are not publicly available due to privacy or ethical restrictions.
